# Dataset and machine learning-based computer-aided tools for modeling working sorption isotherms in dried parchment and green coffee beans

**DOI:** 10.1016/j.dib.2025.111738

**Published:** 2025-05-31

**Authors:** Gentil A. Collazos-Escobar, Andrés F. Bahamón-Monje, Nelson Gutiérrez-Guzmán

**Affiliations:** a Centro Surcolombiano de Investigación en Café (CESURCAFÉ), Departamento de Ingeniería Agrícola, Universidad Surcolombiana, Neiva-Huila, 410001, Colombia; b Grupo de Análisis y Simulación de Procesos Agroalimentarios (ASPA), Instituto Universitario de Ingeniería de Alimentos–FoodUPV, Universitat Politècnica de València, Camí de Vera s/n, Edificio 3F, València, 46022, Spain; c Departamento de Ingeniería Agroindustrial, Facultad de Ingeniería, Universidad Surcolombiana, Neiva, Huila, Colombia

**Keywords:** Hygroscopicity, Water sorption process, Supervised machine learning modeling, Chemometric-based modeling, Artificial intelligence, Storage optimization, Real-time moisture monitoring

## Abstract

This work presents a comprehensive dataset on working sorption isotherms and mid-infrared spectra for parchment husk, parchment coffee, and green coffee beans (*Coffea arabica* L.). The working sorption isotherms were experimentally determined using the Dynamic Dewpoint Isotherm (DDI) method, covering typical storage conditions of dried coffee beans in warehouses. These conditions account for a water activity range (a_w_) from 0.1 to 0.9 and temperatures of 25°C, 35°C, and 45°C. Furthermore, the mid-infrared spectra of parchment husk and green coffee were obtained using Attenuated Total Reflectance-Fourier Transform Infrared (ATR-FTIR) spectroscopy as a complementary tool to analyze the role of parchment covering in the water sorption behavior of parchment coffee beans. The dataset also provides computer-aided tools for the mathematical modeling of working sorption isotherms and infrared data of coffee. These tools were developed using MATLAB® R2023a (The MathWorks Inc., Natick, MA, USA) and offer users the ability to model sorption isotherms and analyze infrared spectral data using advanced machine learning techniques. Thereby, the MATLAB scripts implement an automated routine for the calibration and optimization of the Support Vector Machine (SVM) and Random Forest (RF) techniques, enabling the modeling of working sorption isotherms for each coffee type (considering only a_w_ and temperature) and in a multivariate approach (incorporating a_w_, temperature, and coffee type) to predict the equilibrium moisture content (X_e_). Additionally, the MATLAB script for Principal Component Analysis (PCA) enables users to perform advanced chemometric modeling of the coffee spectra. This script provides a latent-variable-based tool for analyzing spectral patterns associated with different coffee types, allowing for robust model-based differentiation of coffee samples using their infrared properties. These models are particularly valuable as digital representations of the coffee storage process and can be used to optimize storage conditions, understand hygroscopic behavior, and ensure moisture-based quality monitoring in parchment and green coffee beans. The experimental dataset, including working sorption isotherms and mid-infrared spectra, is organized into Excel sheets according to experimental conditions and replicates. The MATLAB scripts come with ready-to-use computational instructions for calibrating predictive models, ensuring precise fitting of isotherms and spectral properties. This dataset represents a valuable asset for researchers, coffee producers, and industry stakeholders, providing practical tools for storage optimization, shelf-life determination, and in-depth analysis of water sorption behavior across different coffee processing stages.

Specifications Table

**Table:** 

Subject	Food engineering
Specific subject area	Food technology, Food engineering, Food Science
Type of data	Excel files: Initial characterization of coffee samples, mid-infrared spectra of parchment and green coffee, and working sorption isotherms of parchment, parchment coffee, and green coffee samples. Figure: Experimental working sorption isotherms, mid-infrared spectra, score plot obtained from PCA results, spectral loadings from the PCA model, and SVM-RF results in the mathematical modeling of isotherms. MATLAB files: PCA modeling, SVM-RF modeling, and the automatic SVM-RF hyperparameter optimization process.
Data collection	Working sorption isotherms (obtained by DDI method) and mid-infrared spectra (obtained by Attenuated Total Reflectance-Fourier Transform Infrared; ATR-FTIR).
Data source location	The experimental dataset described in this work has been obtained in the Centro Surcolombiano de Investigación en Café (CESURCAFÉ) from the Universidad Surcolombiana, Neiva-Huila, Colombia.
Data accessibility	Repository name: Mendeley Data Data identification number: 10.17632/p39cd74rwv.2 Direct URL to data: https://data.mendeley.com/datasets/p39cd74rwv/2 Dataset citation: Collazos-Escobar, Gentil Andres; Bahamón-Monje, Andrés F.; Gutierrez Guzman, Nelson (2025). Dataset, chemometric, and machine learning tools for modeling water sorption isotherms in dried coffee beans, Mendeley Data, V2, doi: 10.17632/p39cd74rwv.2
Related research article	G. A Collazos-Escobar, N. Gutiérrez-Guzmán, H. A. Váquiro, J. V. García-Pérez, J. A. Cárcel, Analysis of machine learning algorithms for the computer simulation of moisture sorption isotherms of coffee beans, Food and Bioprocess Technology. (2025). https://doi.org/10.1007/s11947-025-03785-x

## 1 Value of the Data

• This dataset provides high-resolution working sorption isotherms and mid-infrared spectral data for parchment husk, parchment coffee beans, and green coffee beans, offering critical insights into their hygroscopic behavior and moisture sorption processes. It enables a deeper understanding of how different coffee processing stages (green and parchment) influence moisture sorption and a_w_, which are essential variables for optimizing storage conditions and maintaining coffee quality.
• This dataset is of great significance for food scientists, agricultural engineers, and coffee producers who aim to model the water sorption behavior of dried coffee beans. It serves as a reliable reference for developing predictive models, enhancing the accuracy of storage optimization, and supporting research on shelf-life prediction, packaging materials, and humidity control strategies.
• Mid-infrared spectra obtained through ATR-FTIR spectroscopy enable chemometric analysis, such as PCA, to identify spectral patterns related to moisture behavior in coffee beans. This facilitates the development of explorative models for water sorption during storage.
• The dataset supports mathematical modeling, including supervised machine learning techniques such as SVM and RF, to create a virtual representation of coffee storage process. By integrating a_w_, temperature, and coffee type, these models improve moisture content prediction, aiding real-time decision-making for coffee producers and storage managers.
• By deepening the understanding of moisture dynamics and storage stability in dried coffee, this dataset serves as a valuable tool for sustainable coffee processing. It contributes to the reduction of economic losses, quality control improvements, and enhanced economic viability in coffee production and distribution.
• This dataset also serves as an educational and knowledge transfer resource, as the included MATLAB scripts allow students, researchers, and professionals to explore advanced modeling techniques (e.g., SVM, RF and PCA) using real-world agro-industrial data. This feature promotes hands-on learning, scientific reproducibility, and skill development in the application of artificial intelligence to food and agricultural engineering.

## 2 Background

Coffee is one of the most significant agricultural commodities globally, not only due to its economic value but also because of its sensory attributes and health benefits [1]. Post-harvest processing plays a critical role in determining the quality of the final coffee beverage [2], with drying and storage being two of the most vital steps in preserving its chemical integrity and sensory properties [3]. In the Colombian coffee industry, coffee is typically stored in two primary forms: parchment coffee, where the beans remain encased in their protective endocarp layer after drying, and green coffee, where the parchment layer is removed prior to storage [4]. Despite both forms being stored under similar environmental conditions, their hygroscopic behaviors may differ, potentially influencing moisture migration, shelf life, and the overall stability of coffee during storage [5].

Water sorption isotherms describe the equilibrium relationship between moisture content and a_w_ at a given temperature, providing crucial insights into how food materials interact with their storage environment [6]. These isotherms are essential for predicting moisture changes, ensuring optimal storage conditions, and minimizing deterioration risks, including microbial growth, oxidation, and loss of volatile compounds [7]. Mathematical modeling of sorption isotherms allows for the prediction of moisture equilibrium under varying conditions, offering valuable criteria for optimizing storage protocols and selecting appropriate packaging materials [8]. Traditionally, sorption isotherms have been modeled using empirical equations such as Smith, Peleg, and Halsey, or theoretical models like BET (Brunauer–Emmett–Teller) and GAB (Guggenheim-Anderson-de Boer), where model parameters characterize the physicochemical interactions of water within the coffee matrix [9]. However, current processes within the framework of Industry 4.0 are inherently multivariate, involving vast volumes of data and numerous process variables [10]. As a result, traditional models have limitations, as they primarily describe the influence of a_w_ and temperature on X_e_. In this context, machine learning facilitates the mathematical modeling of the water sorption process in a multivariate manner [11]. By leveraging these techniques, not only can a_w_ and temperature be considered, but additional variables such as coffee bean type can also be integrated. Given the high hygroscopicity of coffee and its exposure to varying humidity levels in storage environments, it is crucial to develop predictive models that include more variables than a_w_ and temperature.

## 3 Data Description

The experimental dataset was compiled in three Excel files; each one is described below.

**InitialCharacterizationCoffee:** This Excel file contains a structured dataset detailing thee initial characterization of different coffee types, specifically parchment husk, parchment coffee, and green coffee. It is organized into five columns, each capturing essential experimental parameters. The first column identifies the coffee type, classifying the data into the three aforementioned groups. The second column corresponds to the sample ID where each coffee type is represented by nine samples (n=9 samples per type of coffee), allowing for a comprehensive analysis of variability. The third column denotes the replicates, as each sample undergoes three independent measurements, ensuring statistical robustness and reproducibility of results. These replicates were included to capture the natural variability within each coffee type, ensuring robust and statistically reliable results. The inclusion of nine replicates per coffee type is statistically sufficient to estimate central tendencies and variability with confidence, allowing for meaningful comparisons [12].

The fourth column provides the moisture content (% wet basis; %w.b.) for each sample. Finally, the fifth column records the a_w_ of each replicate, key parameter which reflects the equilibrium state of water availability within the coffee matrix and its critical value for assessing microbial stability, storage conditions, and shelf-life [13].

**Fig. 1:**
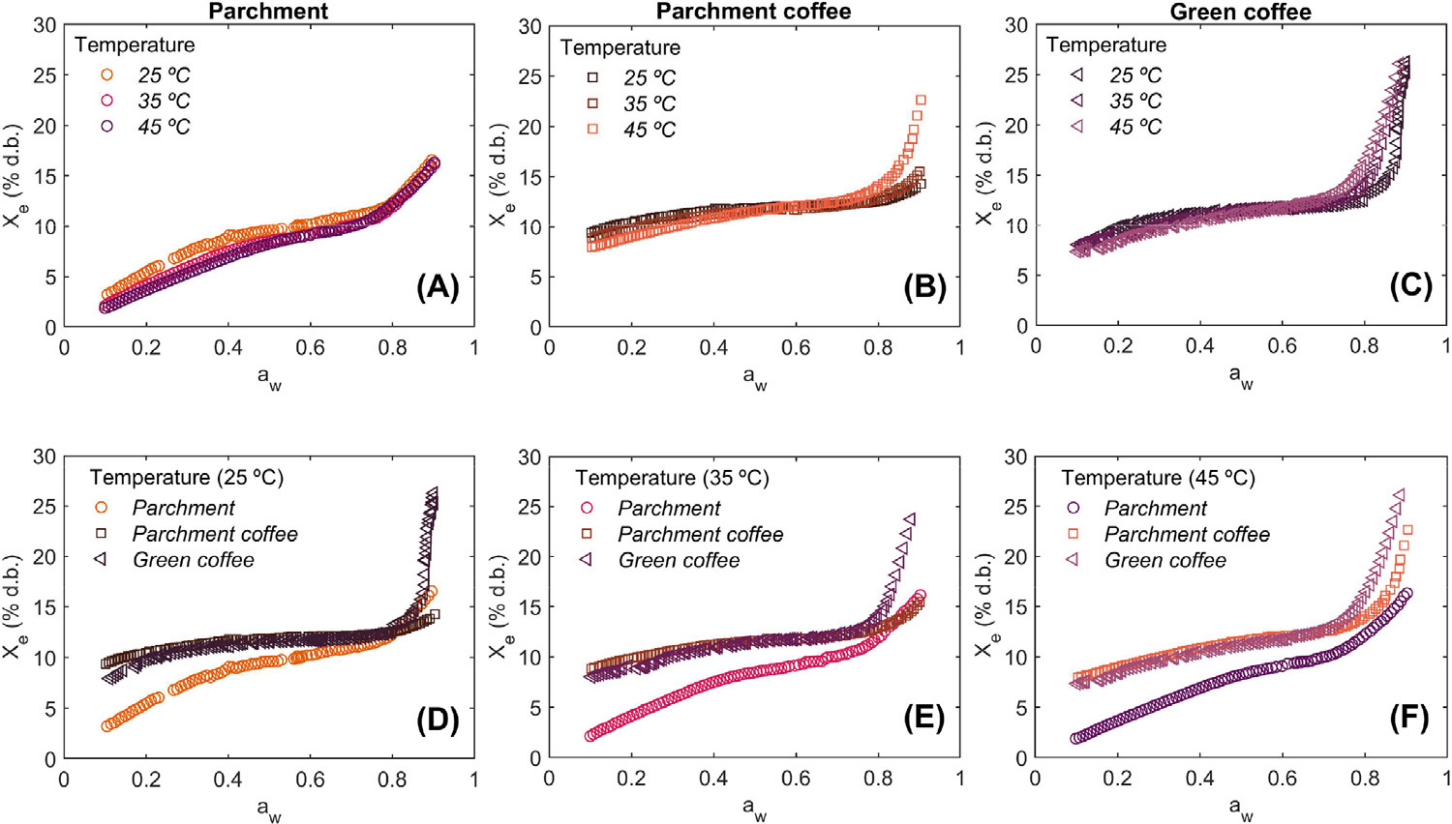
Working sorption isotherms of parchment coffee, parchment, and green coffee at different temperatures. (A) Sorption isotherms of parchment coffee at 25°C, 35°C, and 45°C. (B) Sorption isotherms of parchment at 25°C, 35°C, and 45°C. (C) Sorption isotherms of green coffee at 25°C, 35°C, and 45°C. (D) Comparison of sorption isotherms of parchment, parchment coffee, and green coffee at 25°C. (E) Comparison of sorption isotherms of parchment, parchment coffee, and green coffee at 35°C. (F) Comparison of sorption isotherms of parchment, parchment coffee, and green coffee at 45°C.

**WorkingSorptionIsotherms:** This dataset represents a working sorption isotherms matrix, systematically organized to capture the equilibrium moisture behavior of coffee samples under varying conditions. It consists of 2638 sorption data pairs, each detailing the relationship between a_w_ and X_e_ under controlled temperature conditions (Fig. 1). The first column identifies the sample ID, specifying the individual coffee sample analyzed. The second column denotes the replicate number, ensuring statistical validation and reproducibility of the measurements. The third column categorizes the sorption process into either desorption or adsorption, distinguishing between moisture release and uptake dynamics (see EXPERIMENTAL DESIGN, MATERIALS AND METHODS section). The fourth column compiles the experimental temperature, set at three distinct levels of 25, 35 and 45°C, enabling the study of temperature-dependent hygroscopic behavior. The fifth column contains the corresponding a_w_ values, the sixth column provides the equilibrium moisture content (%dry basis), quantifying the moisture content in samples relative to their dry mass. The final column reports the equilibrium moisture content (%wet basis), expressing moisture content relative to the total sample mass. This dataset serves as a high-resolution working sorption isotherm matrix, allowing for the characterization of moisture equilibrium properties essential for optimizing storage stability, processing conditions, and predictive modeling of coffee hygroscopicity.

The experimental sorption isotherms of parchment (Fig. 1A), parchment coffee beans (Fig. 1B), and green coffee beans (Fig. 1C) exhibited the typical S-shaped Type II curve according to the BET classification.

As a_w_ increased, so did X_e_, and higher temperatures resulted in lower X_e_ values within the a_w_ range 0.1 to 0.7 (Fig. 1). Above 0.7 a_w_, a crossover of the isotherm trends was observed [14]. Additionally, differences in water sorption were evident when comparing parchment, parchment coffee beans, and green coffee beans at the same experimental temperatures (Figs. 1D–1F).

**Mid_InfraredSpectraCoffee:** The dataset consists of raw mid-infrared ATR-FTIR spectra of green coffee and parchment coffee, carefully structured to clarify the individual spectral contributions of each component to the overall hygroscopic behavior. Parchment coffee, being a composite system in which green coffee beans are enclosed within a parchment husk, was analyzed by characterizing each component separately. This approach enabled the assessment of their respective functional groups associated with their roles in moisture sorption process. This understanding is particularly relevant for post-harvest management, as both parchment and green coffee beans are typically stored and handled together.

**Fig. 2:**
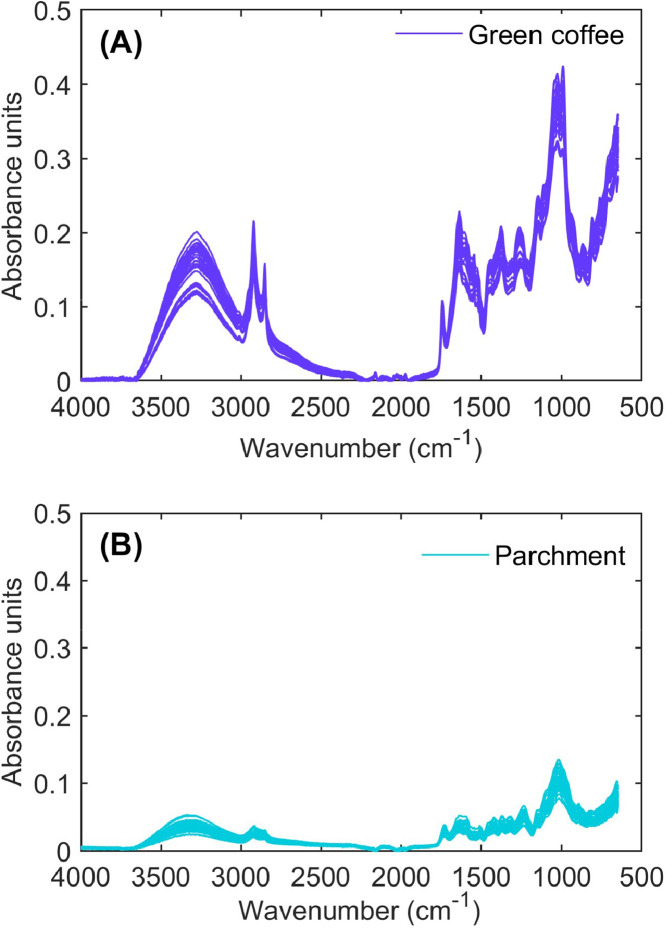
Fourier-Transform mid-infrared (FTIR) spectra of green coffee and parchment. (A) FTIR spectra of green coffee in the wavenumber range of 4000-650 cm^–1^, plotted against absorbance units. (B) FTIR spectra of parchment in the wavenumber range of 4000-650 cm^–1^, plotted as a function of absorbance units.

The dataset is structured across three sheets:

**i) Raw_spectra Green Coffee:** This sheet contains the raw mid-infrared spectra of green coffee samples (Fig. 2A). The first column lists the wavenumber (cm⁻¹), which remain constant across all spectra. Columns 2 to 4 correspond to the three replicate spectra for the first green coffee sample, columns 5 to 7 contain the triplicate spectra for the second sample, and this pattern continues until column 28, resulting in a total of 27 ATR-FTIR spectra, derived from 9 distinct green coffee samples, each measured in triplicate.

**ii) Raw_spectra Parchment Coffee:** This sheet follows the same structure as the first but for parchment husk (Fig. 2B), allowing for direct spectral comparisons between the green coffee and its surrounding parchment layer.

**iii) SpectraldatasetForPCA:** This sheet is designed to facilitate multivariate statistical analysis, particularly chemometric techniques such as PCA for spectral pattern recognition and classification. The first column denotes the coffee type (either parchment or green coffee), while columns 2 to 901 represent wavenumbers spanning the infrared spectral range. The subsequent columns contain absorbance values, where each row corresponds to an individual spectrum, enabling high-dimensional data analysis for model calibration and feature extraction. This dataset is crucial for understanding the molecular composition of coffee at different processing stages, enabling spectroscopic fingerprinting of hygroscopic behavior.

**Fig. 3:**
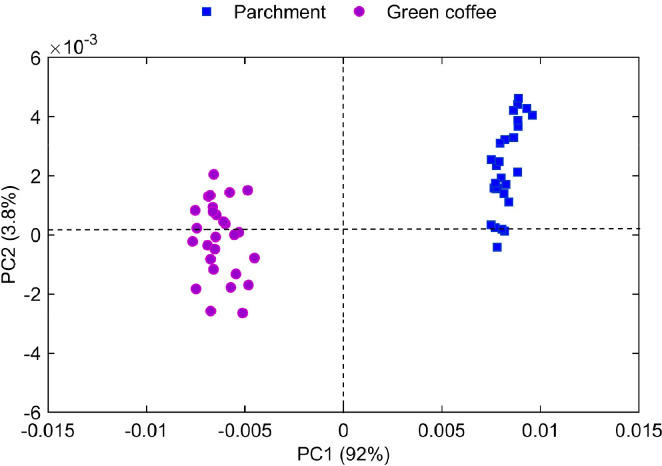
Principal Component Analysis (PCA) score plot of Fourier-transform mid-infrared (ATR-FTIR) spectra. The plot shows the scores of the first principal component (PC1) versus the second principal component (PC2), with the mid-infrared spectra of parchment husk and green coffee projected onto the components.
vi. **Loadings plot:** This plot (Fig. 4) shows the contribution of each spectral variable (wavenumber) to the first two principal components. The loadings represent how strongly each spectral feature influences the components, with high absolute values indicating significant influence. This figure is crucial for interpreting which specific spectral features drive the observed variation in the data.

**PrincipalComponentAnalysisModeling:** This MATLAB script applies PCA model to a spectral dataset of coffee samples. The user is prompted to input the number of principal components to extract from the dataset. The dataset, located in the third sheet of the **Mid_InfraredSpectraCoffee.xlsx** file, contains mid-infrared spectra of green and parchment coffee, organized by wavenumber and absorbance data. The maximum number of principal components that can be extracted is calculated as the minimum between the number of spectra and wavenumbers, minus one. This number is displayed in the MATLAB command window for the user’s reference. Once the user specifies the number of principal components (NLVs), PCA is performed using Singular Value Decomposition (SVD) with mean-centering enabled. This yields principal component scores, loadings, explained variance, and other statistics related to the reconstruction of the data. Various plots are then generated to assist in visualizing and interpreting the results of the PCA, which are outlined as follows:

i. **Explained variance bar plot:** This bar plot shows the percentage of variance captured by each principal component. Since PCA orders the components by the amount of variance they explain, this plot allows for assessing the importance of each PC. The higher the variance, the more informative the principal component is for representing the data.
ii. **Cumulative explained variance.** This plot displays the cumulative variance explained as more components are added. It helps determine how many principal components should be retained to achieve an adequate representation of the data. Typically, the cumulative variance increases sharply at first and then flattens, indicating the point where additional components no longer contribute significant new information.
iii. **Residual Sum of Squares (RSS):** This plot illustrates the residual variance left after reconstructing the data using the selected principal components. The RSS measures how well the selected components represent the original data. Statistical thresholds (95^th^, 97.5^th^, and 99^th^ percentiles) are drawn to help identify observations with high residual error, which may suggest outliers or poorly fitted data points.
iv. **Hotelling’s T-Squared (T^2^):** This plot shows Hotelling’s T² statistic, which measures how much each observation deviates from the central distribution of the data in the principal component space. Observations significantly far from the center are potential anomalies, and the plot includes percentile thresholds (95^th^, 97.5^th^, and 99^th^) to help flag these outliers.
v. **Score plot:** The score plot (Fig. 3) visualizes the distribution of samples in the reduced dimensional space, projected onto the first two principal components (PC1 and PC2). This allows for identifying patterns or clusters in the data, with different markers or colors representing different coffee types (parchment husk or green coffee). The plot provides insight into the relationships between the samples based on their spectral characteristics.

**Fig. 4:**
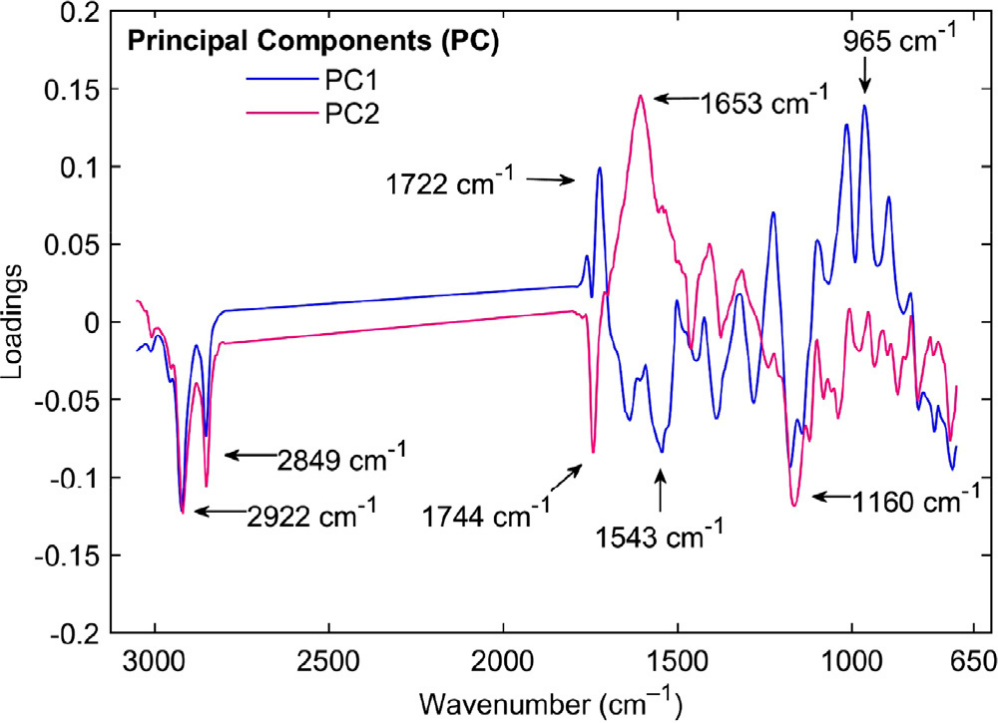
Principal Component Analysis (PCA) loadings versus wavenumber (cm^–1^). The loadings for the first (PC1) and second (PC2) principal components are plotted against the wavenumber. The analysis identifies the functional groups in the Fourier-transform mid-infrared (ATR-FTIR) spectra that contributed to the differentiation between parchment husk and green coffee.

By offering these visualizations, the script helps users better understand the underlying patterns and variability in the spectral data, enabling informed decisions for further analysis or data interpretation.

**SupportVectorMachineLearningModeling_Coffee:** This MATLAB script is designed to SVM regression modeling on working sorption isotherm data obtained from coffee samples. The modeling can be executed in two different modes: either by fitting separate models for each type of coffee (parchment husk, parchment coffee, or green coffee), or by training a single multivariate model that includes coffee type as one of the input features. The main idea is to optimize and assess SVM model performance for predicting equilibrium moisture content based on selected input features using a randomized hyperparameter search and standard performance metrics. The MATLAB code begins by preparing the environment, clearing variables, closing figures, and importing experimental data from an Excel file **WorkingSorptionIsotherms**, while setting a fixed random seed to ensure reproducibility of results. The user is then prompted to choose between two modeling strategies: (1) modeling each coffee type separately, where the dataset is filtered to include only the selected type of coffee and the model is trained using only a_w_ and temperature as regressors and the X_e_ as response variable, or (2) using a multivariate approach where all coffee types are combined and the type itself is encoded as an additional numeric feature, allowing the model to learn patterns across coffee types. In every case, the data is split into training and validation datasets ensuring a 75%-25% random split, respectively. A key highlight of the script is its use of random search for hyperparameter optimization, a method that balances efficiency and performance by sampling a defined hyperparameter space at random. The script assesses 50 unique combinations of the regularization parameter (C; ranging from 0.1 to 100), epsilon values (from 0.01 to 0.5), and kernel functions (“Linear”, “Gaussian”, and “Polynomial”).

Compared to the grid search method (commonly used in hyperparameter optimization of machine learning techniques), which exhaustively evaluates every possible combination in a structured way and becomes computationally expensive as the number of hyperparameters increases, random search is more practical in higher-dimensional spaces, often identifying near-optimal or even superior parameter sets in fewer iterations by covering the space more broadly and unpredictably. In any case, the MATLAB code allow the users to incorporate for instance more kernel functions and/or modified the C and epsilon values. For each sampled configuration, an SVM regression model is trained, and performance is assessed on both training and validations datasets using root mean squared error (RMSE; %d.b., Eq. (1)). All results are stored, and the model with the lowest test RMSE is selected as the optimal model. After training, the script computes several goodness of fit metrics such as mean relative error (MRE; %, Eq. (2)), and coefficient of determination (R^2^; %, Eq. (3)).

(1) $$\text{RMSE}\;\left(\%d.b.\right)=\sqrt{\frac{\sum_{i=1}^{n}{\left(X_{e\;}-X_{\text{epred}}\right)}^{2}}{n}}$$

(2) $$\text{MRE}\;\left(\%\right)=\frac{100}{n}\sum_{i=1}^{n}\frac{\left|X_{e\;}-X_{\text{epred}}\right|}{X_{e}}$$

(3) $$R^{2}\left(\%\right)=100-\frac{\sum_{i=1}^{n}{\left(X_{e\;}-X_{\text{epred}}\right)}^{2}}{\sum_{i=1}^{n}{\left(\bar{X_{e\;}}-\;X_{\text{epred}}\right)}^{2}}$$

where n is the number of experimental dataset.

**Fig. 5:**
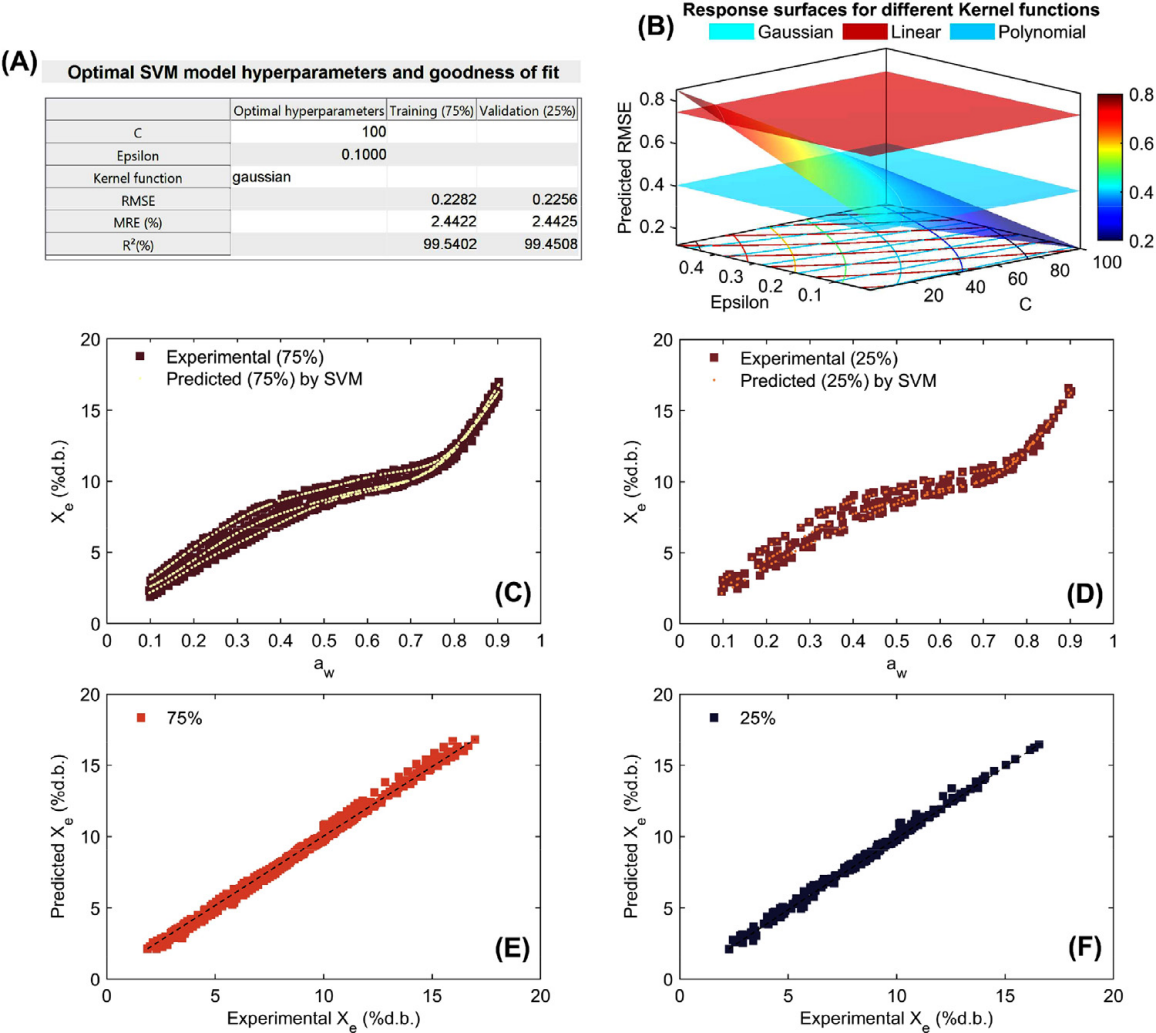
Optimization and performance assessment of the trained and validated Support Vector Machine (SVM) technique for modeling working sorption isotherms in parchment husk. The results highlight the best-performing hyperparameters, including C, Epsilon, and the Kernel function, along with the corresponding metrics: root mean square error (RMSE), mean relative error (MRE%;), and coefficient of determination (R^2^; %) for both the training (75%) and validation (25%) datasets (A). Response surface methodology was employed in the grid search process to optimize the SVM hyperparameters, minimizing RMSE by selecting the optimal values for C, Epsilon, and the Kernel function (B). Experimental vs. SVM-predicted working sorption isotherms for the training dataset (C) and validation dataset (D), and linear agreement between experimental and SVM-predicted equilibrium moisture content (Xe) for both the training (E) and validation (F) datasets.

**Fig. 6:**
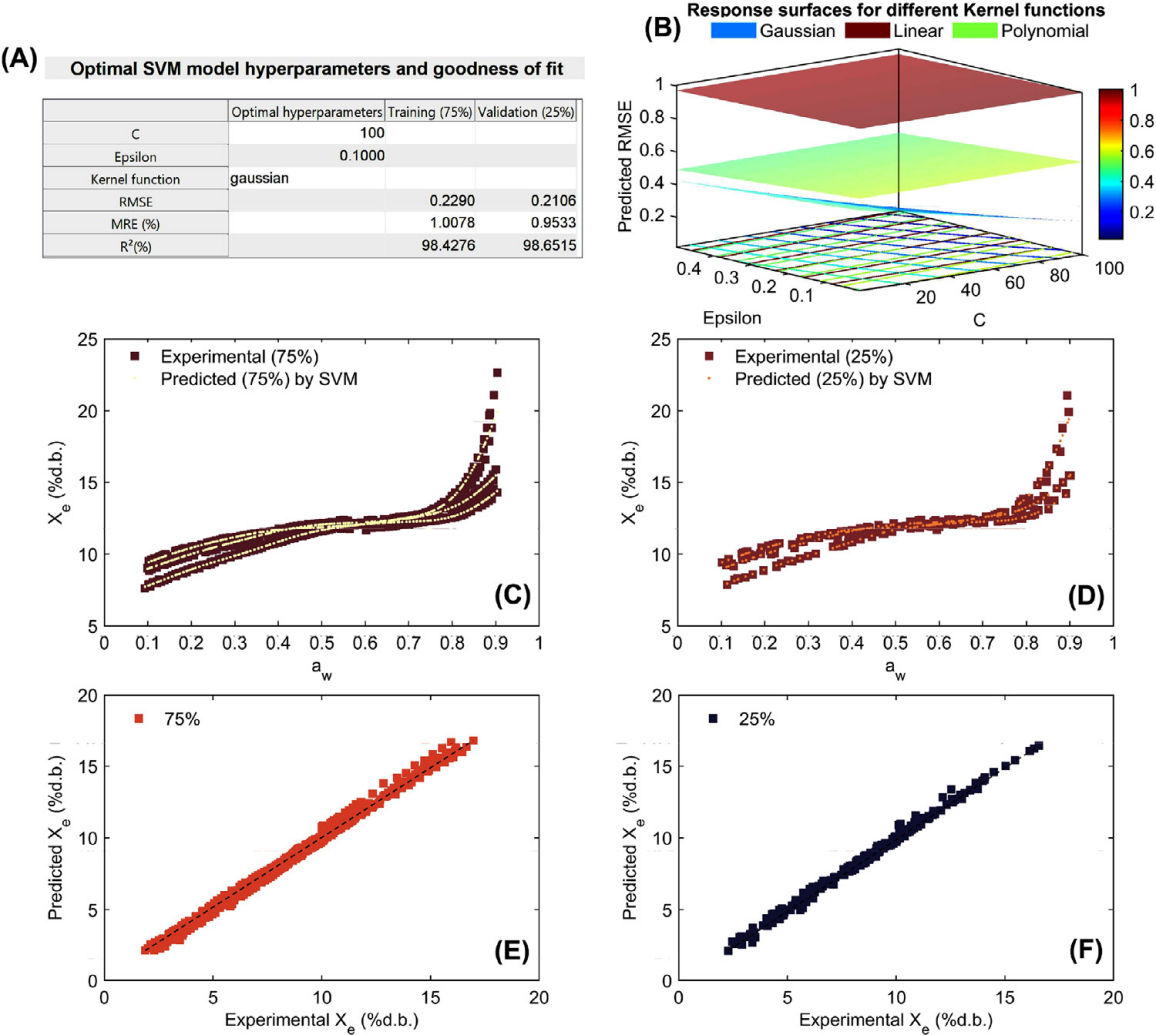
Optimization and performance assessment of the trained and validated Support Vector Machine (SVM) technique for modeling working sorption isotherms in parchment coffee beans. The results highlight the best-performing hyperparameters, including C, Epsilon, and the Kernel function, along with the corresponding metrics: root mean square error (RMSE), mean relative error (MRE%;), and coefficient of determination (R^2^; %) for both the training (75%) and validation (25%) datasets (A). Response surface methodology was employed in the grid search process to optimize the SVM hyperparameters, minimizing RMSE by selecting the optimal values for C, Epsilon, and the Kernel function (B). Experimental vs. SVM-predicted working sorption isotherms for the training dataset (C) and validation dataset (D), and linear agreement between experimental and SVM-predicted equilibrium moisture content (Xe) for both the training (E) and validation (F) datasets.

**Fig. 7:**
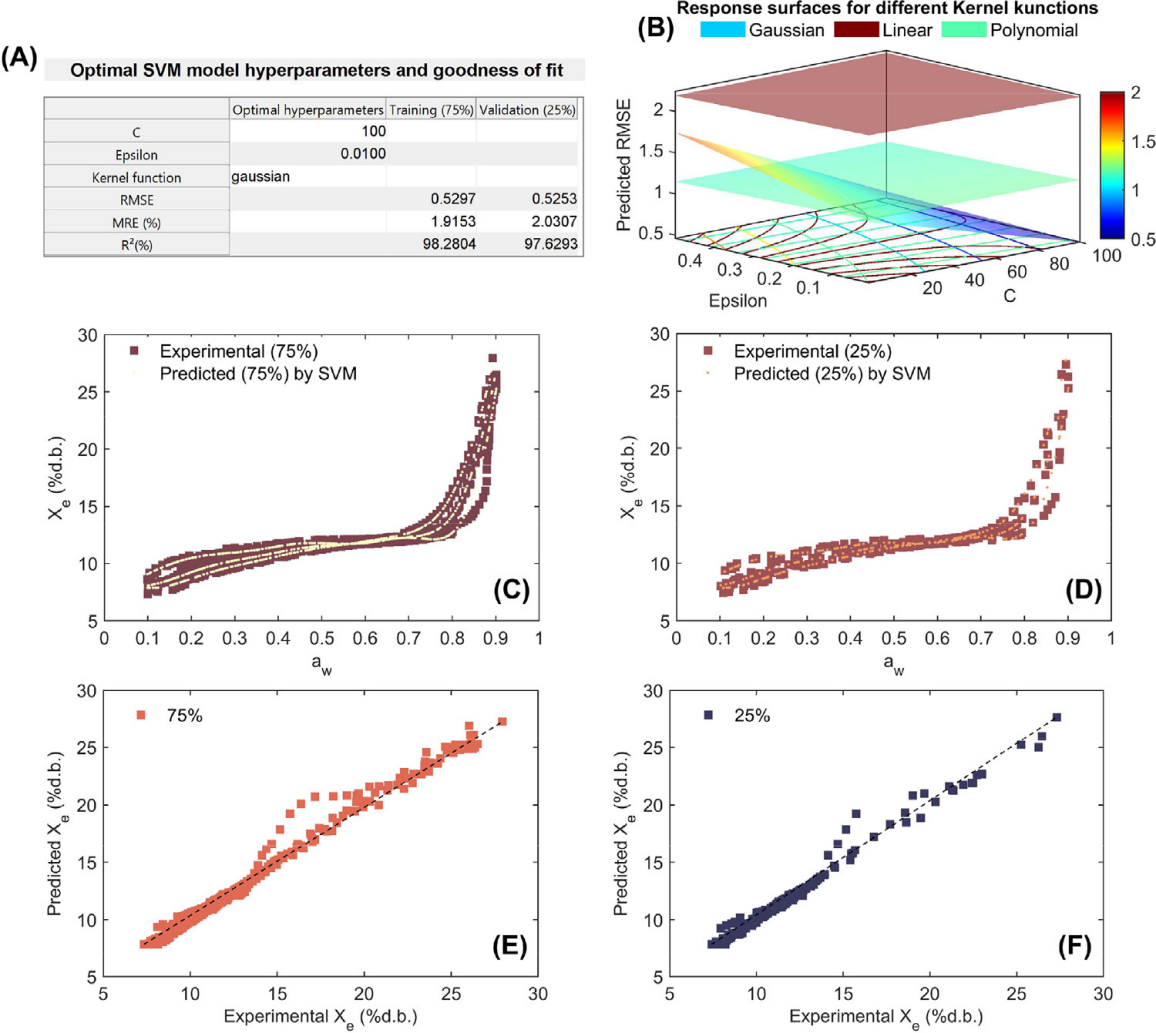
Optimization and performance assessment of the trained and validated Support Vector Machine (SVM) technique for modeling working sorption isotherms in green coffee beans. The results highlight the best-performing hyperparameters, including C, Epsilon, and the Kernel function, along with the corresponding metrics: root mean square error (RMSE), mean relative error (MRE%;), and coefficient of determination (R^2^; %) for both the training (75%) and validation (25%) datasets (A). Response surface methodology was employed in the grid search process to optimize the SVM hyperparameters, minimizing RMSE by selecting the optimal values for C, Epsilon, and the Kernel function (B). Experimental vs. SVM-predicted working sorption isotherms for the training dataset (C) and validation dataset (D), and linear agreement between experimental and SVM-predicted equilibrium moisture content (Xe) for both the training (E) and validation (F) datasets.

**Fig. 8:**
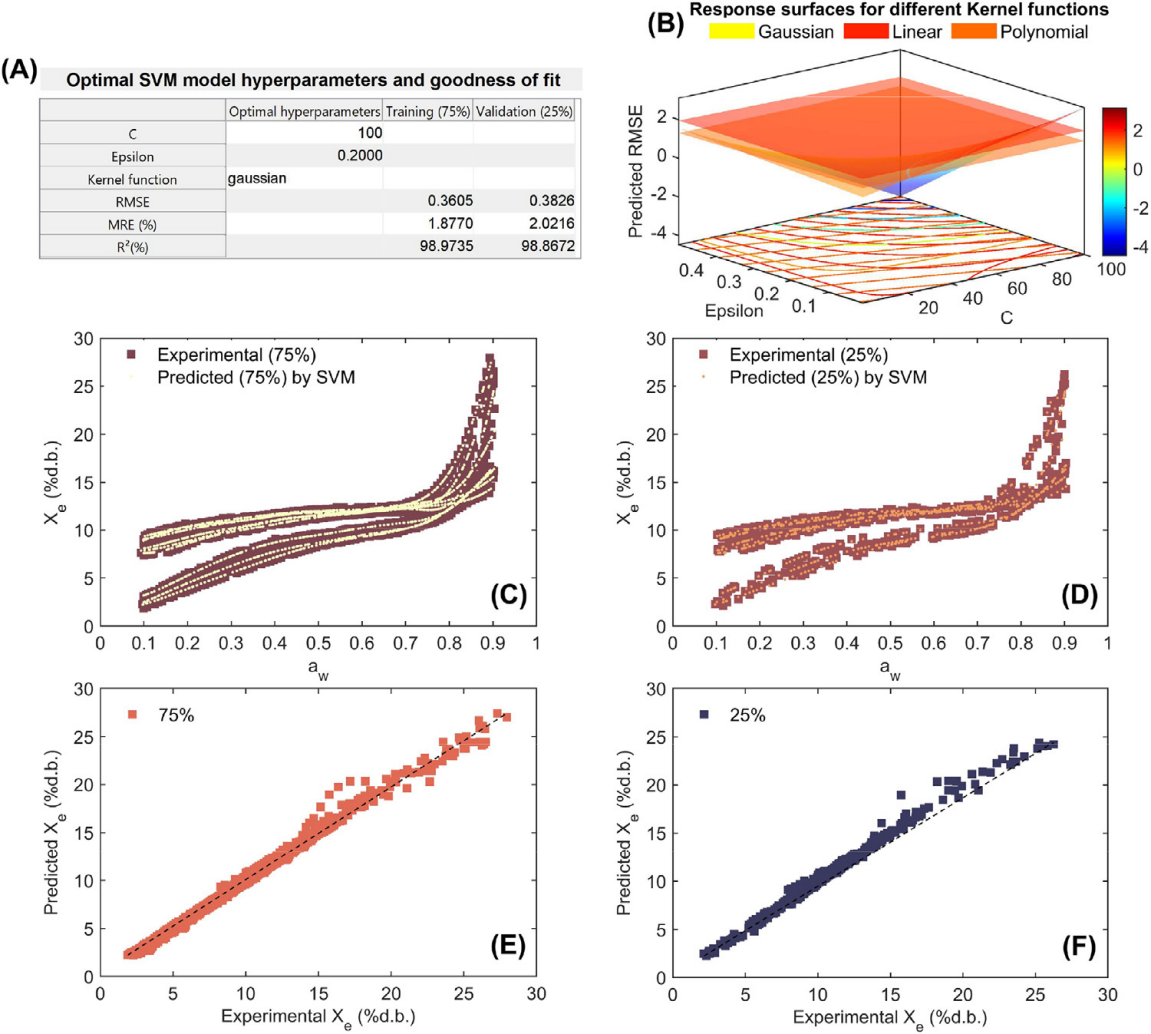
Optimization and performance assessment of the trained and validated Support Vector Machine (SVM) technique for modeling working sorption isotherms across all coffee types. The results highlight the best-performing hyperparameters, including C, Epsilon, and the Kernel function, along with the corresponding metrics: root mean square error (RMSE), mean relative error (MRE%;), and coefficient of determination (R^2^; %) for both the training (75%) and validation (25%) datasets (A). Response surface methodology was employed in the grid search process to optimize the SVM hyperparameters, minimizing RMSE by selecting the optimal values for C, Epsilon, and the Kernel function (B). Experimental vs. SVM-predicted working sorption isotherms for the training dataset (C) and validation dataset (D), and linear agreement between experimental and SVM-predicted equilibrium moisture content (Xe) for both the training (E) and validation (F) datasets.

Finally, the script generates a set of visual outputs including a summary table of the optimal parameters and performance metrics, parity plots comparing experimental X_e_ vs. predicted (X_epred_) values for training and validation datasets, and 3D response surface plots that illustrate how RMSE varies with C and epsilon across the different kernel functions. These surfaces help interpret the influence of hyperparameters on model quality and offer intuitive insight for fine-tuning. Additionally, standardization is internally handled within the SVM model to ensure consistent feature scaling, which is especially important for kernel-based methods. The statistical results for the mathematical modeling of working sorption isotherms are presented in Fig. 5, Fig. 6, Fig. 7, Fig. 8.

As can be seen in Fig. 5, Fig. 6, Fig. 7, Fig. 8, the high ability of SVM regression model in the mathematical description of working sorption isotherms in all types of coffee and for both training (75%) and validation (25%). Statistical results showed that SVM accurately (low RMSE, MRE<10% and R^2^>97%) described the influence of a_w_ and temperature on X_e_ (independent modeling approach; Figs. 5A-7A) per each type of coffee. Furthermore, the SVM-modeling approach considering the a_w_, temperature and type of coffee (parchment husk, parchment coffee beans and green coffee beans) on X_e_ was also successful due to the high goodness of fit metrics (RMSE=0.361%d.b., MRE=1.877% and R^2^=98.973% for training and RMSE=0.382%d.b., MRE=2.022% and R^2^=98.867% for validation, Fig. 8A). Regarding the optimization parameters, the “Gaussian” kernel function in all cases exhibited the lowest RMSE across a wide range of hyperparameters, reinforcing its suitability for this dataset (Figs. 5B-8B). As a result, a high correspondence between experimental and predicted working sorption isotherms (Figs. 5C-F to 8C-F) was found by using the optimized SVM model.

Complementary to the SVM machine learning modeling, an additional technique was also included in the analysis of sorption isotherms.

**Fig. 9:**
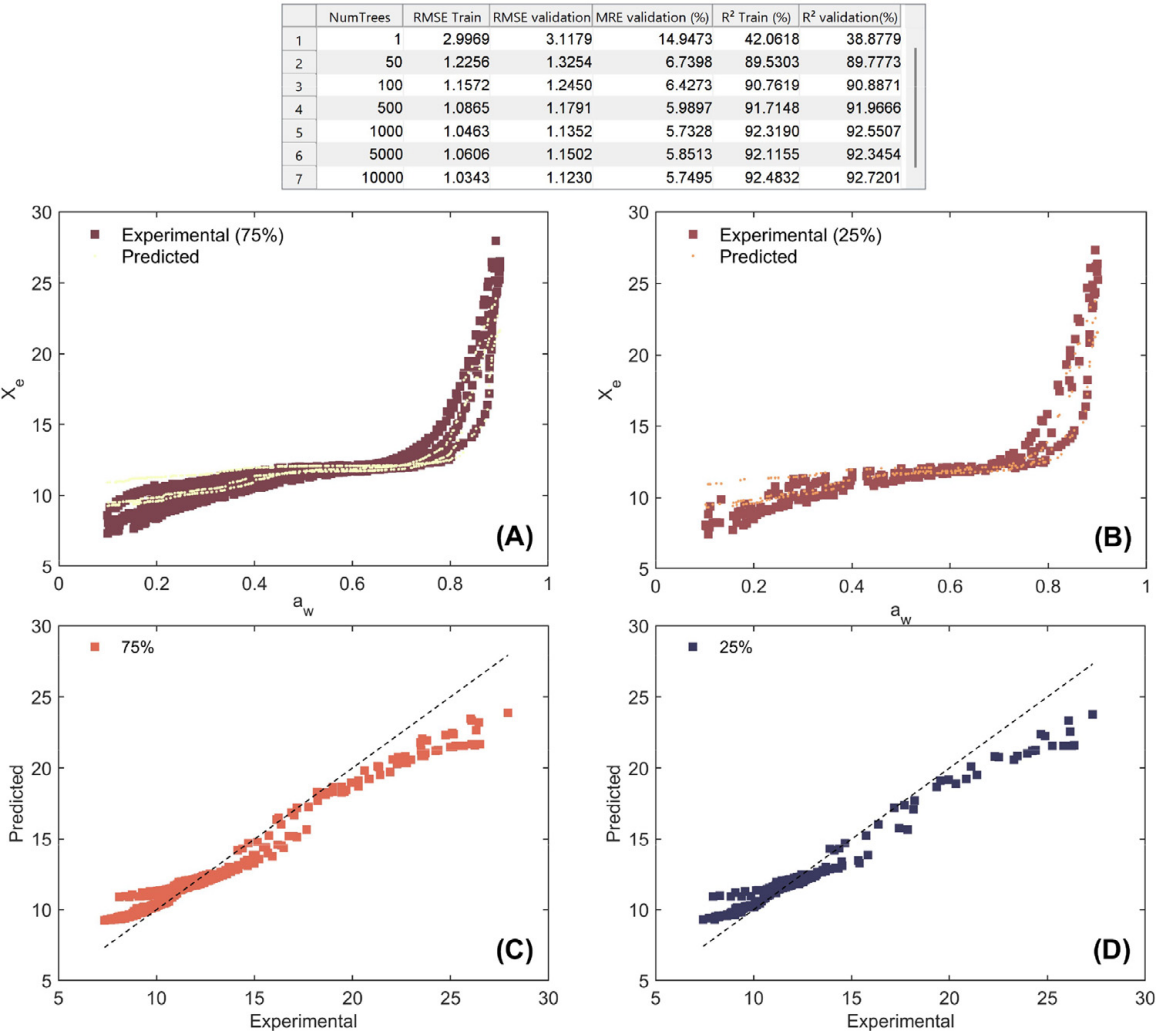
Optimization and performance assessment of the trained and validated Random Forest (RF) technique for modeling working sorption isotherms across all coffee types. The results highlight the best-performing hyperparameters, including different number of trees, along with the corresponding metrics: root mean square error (RMSE), mean relative error (MRE), and coefficient of determination (R^2^) for both the training (75%) and validation (25%) datasets. Experimental vs. RF-predicted working sorption isotherms for the training dataset (A) and validation dataset (B), and linear agreement between experimental and RF-predicted equilibrium moisture content (X_e_) for both the training (C) and validation (D) datasets.

**RandomForestLearningModeling_Coffee**: This MATLAB script is designed for RF regression modeling on working sorption isotherm data obtained from coffee samples. The modeling can be performed in two distinct modes: either by fitting independent models for each type of coffee material (parchment husk, parchment coffee, or green coffee), or by training a unified multivariate model that incorporates coffee type as an additional predictor. The primary goal is to optimize and evaluate the predictive performance of RF models for estimating X_e_ based on selected input features such as a_w_, temperature, and optionally, coffee type. The script begins by preparing the MATLAB environment by clearing variables, closing figures, and importing experimental data from the Excel file **WorkingSorptionIsotherms**. A fixed random seed is set to ensure reproducibility of the modeling results. The user is prompted to select one of the two modeling approaches: (1) building separate models for each coffee type, using only a_w_ and temperature as regressors and X_e_ as the response variable, or (2) constructing a multivariate model that includes all coffee types by encoding the coffee type numerically and using it as a third input feature. In both approaches, the dataset was randomly divided into training (75%) and validation (25%) dataset. For each configuration, the script fits RF models by varying the number of decision trees (NumTrees) from a user-defined list. For each model, key performance metrics are computed including RMSE (Eq. (1)), MRE (Eq. (2)), and R^2^ (Eq. (3)) for both training and validation datasets. These metrics allow the user to assess model generalization and avoid overfitting. Unlike hyperparameter tuning through random search or grid search, this version of the script focuses on systematically assessing the impact of the NumTrees on prediction performance. By iterating through different amount of random trees, the model’s bias-variance balance was explored. All results are stored in a summary table showing performance metrics for each configuration (Fig. 9), allowing for easy comparison and selection of the best model based on lowest RMSE or highest R^2^.

Both SVM and RF statistical procedures were conducted by using non-linear Ordinary Least Square (OLS) regression method and statistical parameter estimation was carried out using MATLAB programming environment.

Future works should be conducted to implement the developed models as digital representations of coffee storage under real industrial conditions. This includes integrating the predictive models into sensor-based monitoring systems for real-time moisture control or embedding them into decision-support tools for storage facility management. Furthermore, the dataset could be expanded with additional variables (e.g., analysis sensory quality attributes and bioactive compounds in coffee beans during storage) to enhance machine learning model generalizability. These future directions would contribute to the development of smart storage technologies and promote data-driven optimization of post-harvest processes in the coffee industry.

## 4 Experimental Design, Materials and Methods

**Fig. 10:**
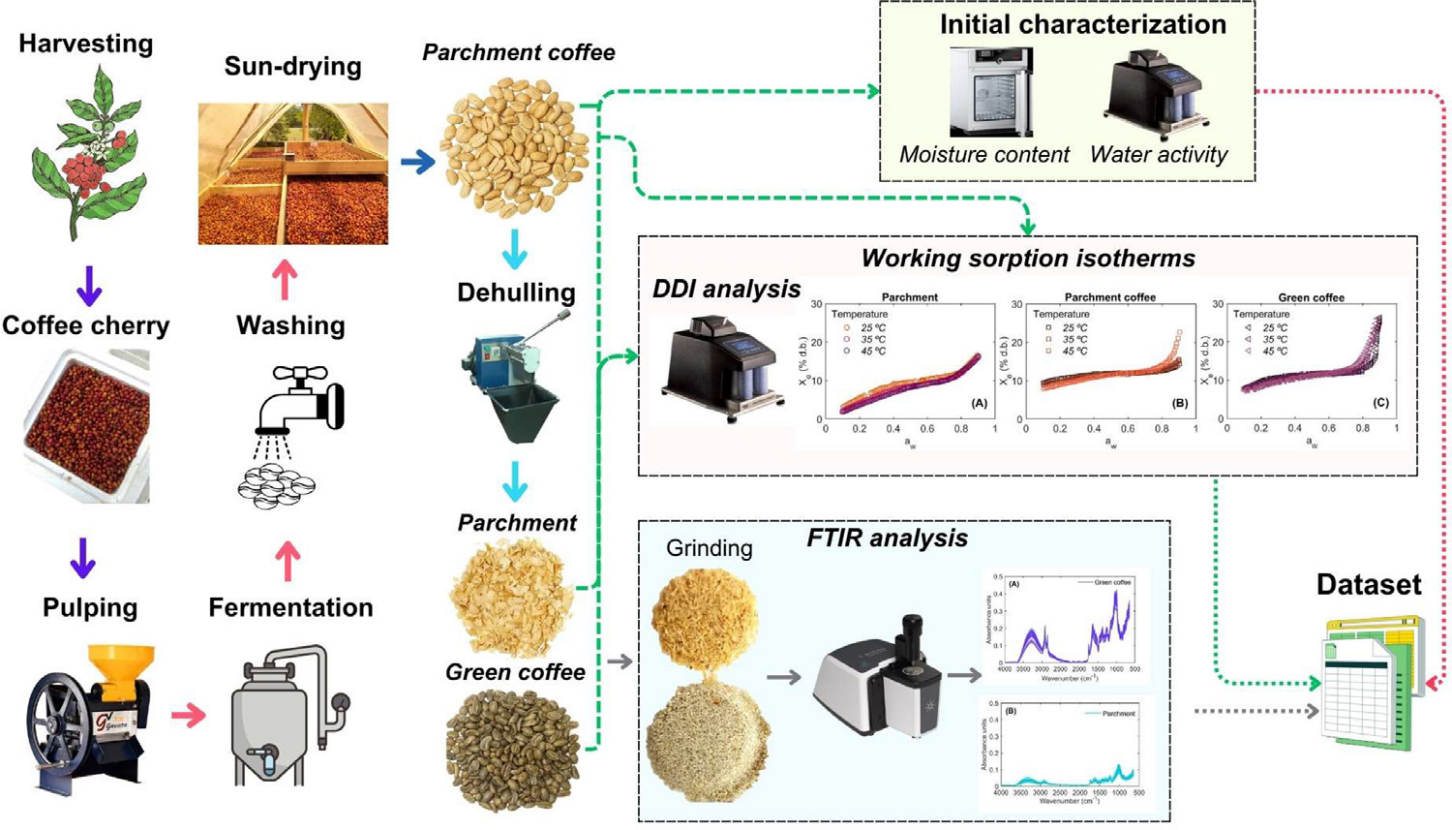
Schematic representation of the experimental workflow for obtaining a dataset on working sorption isotherms and mid-infrared spectral properties of coffee. The process includes sample processing, initial characterization, experimental determination of sorption isotherm and mid-infrared spectral acquisition.

**Fig. 11:**
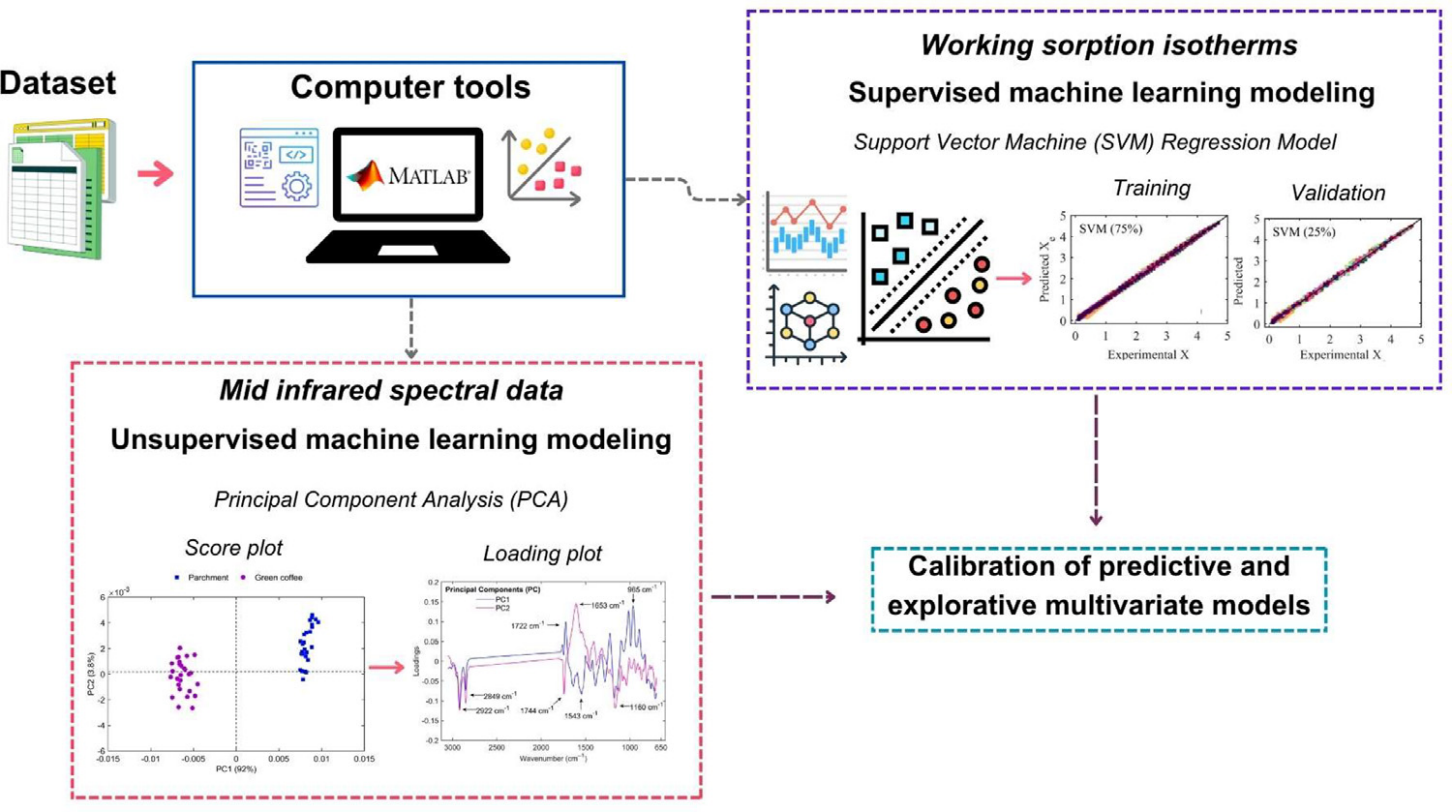
Flowchart of the computer-aided procedure for applying supervised and unsupervised machine learning models to calibrate predictive and exploratory models for working sorption isotherms and mid-infrared spectral properties.

The experimental workflow employed for the processing of coffee samples and the subsequent compilation of the dataset and computational tools is depicted in Fig. 10, Fig. 11, respectively. Fresh coffee cherries (*Coffea arabica* L. var. Colombia; Fig. 10) were manually harvested from various cultivars in the Huila region of Colombia. The selection of coffee cultivars was based on farms certified under the *Good Coffee Farming Practices* program by the National Federation of Coffee Growers of Colombia. This certification ensures that the beans were cultivated under standardized and high-quality agricultural practices. While this approach helps maintain consistency and reliability in sample quality, it also supports the representation of broader characteristics typical of specialty coffee beans in the region [2].

Approximately 30 kg of harvested cherries were transported under refrigerated conditions (4 °C) to the Centro Surcolombiano de Investigación en Café (CESURCAFÉ, Neiva-Huila, Colombia), where they were processed via the wet method. The post-harvest processing involved depulping the cherries using a mechanical pulper (Gaviota 300, Ingesec, Colombia), followed by fermentation in plastic containers for 18 hours. After fermentation, the beans were thoroughly washed to remove residual mucilage. Subsequently, the samples were sun-dried over a period of 9 days, between 9:00 a.m. and 5:00 p.m., under environmental conditions of 33 ± 3 °C and 20–45% relative humidity. During drying process, the moisture content of beans was monitored every hour using a portable grain moisture meter (Kett PM-450, Science of Sensing, Japan) until the beans reached a target moisture content of 9–11%w.b. Following drying, the parchment coffee samples were subdivided into two homogeneous batches using a sample divider (Boerner, Magra, Colombia). One batch was allocated for determining working sorption isotherms in parchment coffee beans (Fig. 10), while the second batch underwent hulling using a mechanical huller (ING-C-250, Ingesec, Colombia) to remove the parchment husk and yield green coffee beans, both of which were also used for the determination of working sorption isotherms. This procedure was performed to avoid any bias and ensure all coffee samples were analyzed across all coffee types, making them representative of the entire sample population.

Moisture content of the parchment husk, parchment coffee, and green coffee beans was determined by gravimetric analysis using a precision balance (PB3002-S, Mettler Toledo, USA). Approximately 10 g of each sample was oven-dried at 105 ± 1 °C (UF55, Memmert GmbH + Co. KG, Schwabach, Germany) until a constant weight was achieved (approximately 24 h). Post-drying, samples were cooled in a desiccator for 5 hr and then weighed. The a_w_ was assessed using a vapor sorption analyzer (VSA, Aqualab, Decagon Devices, USA), and all sample measurements were conducted in triplicate. Previous to the a_w_ the VSA equipment was calibrated using different saturated salt solutions of LiCl (13.41 mol/kg, a_w_= 0.250 ± 0.003; 8.57 mol/kg, a_w_=0.500 ± 0.003) and NaCl (6.0 mol/kg, a_w_=0.760 ± 0.003) provided by the instrument manufacturer. To achieve this, saturated salt solutions were individually placed inside the instrument chamber, and the corresponding a_w_ was measured. Each measured a_w_ value was recorded by the instrument and stored as a standard reference for the respective a_w_ level.

Working sorption isotherms of the parchment husk, parchment coffee, and green coffee beans were determined in triplicate using the Dynamic Dewpoint Isotherm (DDI) method with the VSA (Aqualab, Decagon Devices Inc., Pullman, WA, USA). As the samples exhibited intermediate a_w_ values (0.55<a_w_< 0.60 [15]), both desorption (from current a_w_ to 0.1) and adsorption (from current a_w_ 0.6 to 0.9) processes were evaluated. Experiments were performed at three temperatures (25, 35, and 45 °C), using a water activity resolution of 0.01 a_w_ and an airflow rate of 100 mL min^–1^. In this way, a real storage process was imitated, where the moisture level of samples can go down (desorption) or go up (adsorption) based on the surrounding conditions, which helps control the data spread caused by hysteresis [16].

To spectrally characterize the samples and investigate the role of parchment husk in the moisture sorption behavior of coffee beans, Fourier-transform mid-infrared spectroscopy (FTIR) was employed. Spectral data were collected using an FTIR spectrophotometer (Cary 630, Agilent Technologies, USA) equipped with a diamond attenuated total reflectance (ATR) accessory and a ZnSe crystal. Ground parchment husk and green coffee bean samples (Bezzera BB004NR0IL2, Italy) were analyzed at room temperature (20 ± 0.5 °C). Approximately 1 g of each ground sample was placed on the ATR accessory and compressed for optimal contact. Background spectra were acquired from the clean accessory prior to each sample measurement. Spectra were recorded in the mid-infrared region (4000–650 cm^–1^), with a resolution of 8 cm^–1^, a scan rate of 20, and background correction applied. All spectral analyses were performed in triplicate.

After acquiring the dataset containing both working sorption isotherms and mid-infrared spectral data, two machine learning modeling approaches were developed (Fig. 10). The first approach involved the application of supervised learning techniques, specifically SVM and RF regression, for modeling the sorption isotherms of coffee samples.

A dedicated two MATLAB scripts (**SupportVectorMachineLearningModeling_Coffee.m**) and (**RandomForestLearningModeling_Coffee.m)** were implemented to perform SVM and RF regression on experimental data, either by fitting individual models for each coffee component (parchment husk, parchment coffee, or green coffee) or by constructing a multivariate model incorporating coffee type as an additional input variable (see DATA DESCRIPTION section). The second modeling approach consisted of PCA to the mid-infrared spectral data in order to explore the underlying variance structure and to identify spectral patterns associated with different coffee matrices. The MATLAB script (**PrincipalComponentAnalysisModeling.m**) extracted user-defined NLVs using SVD with mean-centering. The analysis produced a series of diagnostic plots, including explained variance, cumulative variance, RSS, T^2^, score plots, and loadings plots, allowing for in-depth interpretation of the spectral differences between parchment husk and green coffee (see DATA DESCRIPTION section).

## Limitations

Not applicable.

## Ethics Statement

The dataset compiled in this study did not involve human subjects, animal experiments, or data obtained from social media platforms.

## CRediT Author Statement

**Gentil A. Collazos-Escobar:** Conceptualization, Methodology, Investigation, Software, Data curation, Visualization, Writing–original draft, Writing–review & editing. **Andrés F. Bahamon-Monje:** Conceptualization, Formal analysis, Investigation, Software, Data curation, Writing–original draft, Writing–review & editing. **Nelson Gutiérrez-Guzmán:** Conceptualization, Funding acquisition, Investigation, Project administration, Resources, Validation, Supervision, Writing- Reviewing and Editing.

## Declaration of Competing Interest

The authors declare that they have no known competing financial interests or personal relationships that could have appeared to influence the work reported in this paper.

## Data Availability

Mendeley DataDataset, chemometric, and machine learning tools for modeling water sorption isotherms in dried coffee beans (Original data)
